# Comparative Evaluation of the Antibacterial Efficacy of Chlorhexidine and 810 nm Diode Laser in the Disinfection of Root Canals Contaminated With Enterococcus faecalis: An In Vitro Study

**DOI:** 10.7759/cureus.28596

**Published:** 2022-08-30

**Authors:** Teris Mathew, Shanthala BM, Prashanth GV, Jubin Jose

**Affiliations:** 1 Department of Pedodontics and Preventive Dentistry, Coorg Institute of Dental Sciences, Coorg, IND; 2 Department of Periodontology, Al-Ameen Dental College, Bangalore, IND; 3 Department of Orthodontics and Dentofacial Orthopaedics, Al Muhallab Dental Center, Kuwait, KWT

**Keywords:** endodontics, enterococcus faecalis, root canal disinfection, chlorhexidine, diode laser

## Abstract

Introduction: This study compared the antibacterial efficacy of three different root canal irrigants, sodium hypochlorite, chlorhexidine and 810 nm diode laser and diode laser in combination with chlorhexidine in root canals contaminated with *Enterococcus faecalis.*

Methods: Fifty extracted mandibular first premolars were decoronated at the cementoenamel junction and cut at the apical end; biomechanical preparation was done, autoclaved and contaminated with *Enterococcus faecalis.* The specimens were divided into five groups containing 10 teeth each: group I: saline (negative control), group II: 2.5% sodium hypochlorite, group III: 2% chlorhexidine gluconate solution, group IV: diode laser, and group V: diode laser in combination with 2% chlorhexidine. Disinfection was carried out, after which dentinal shavings were collected from the specimens of each group. Antimicrobial efficacy was tested by counting the colony-forming units of viable *Enterococcus faecalis* on the agar plates. One-way ANOVA and Scheffe’s post hoc test were done to analyse the results.

Results: Diode laser with chlorhexidine group showed minimum colony-forming units followed by the diode laser group. The post hoc test showed a statistically significant difference between saline, 2.5% sodium hypochlorite and 2% chlorhexidine groups (P = 0.001) and a non-significant difference between diode laser and diode laser with chlorhexidine group (P = 0.997).

Conclusions: Diode laser can be used as a root canal disinfectant alone or in combination with chlorhexidine.

## Introduction

Over the years, root canal disinfectants have been studied in great detail. Sodium hypochlorite is often considered a gold standard due to its antibacterial and tissue-dissolving properties [1]. However, irritation to periapical tissues, unpleasant taste, high toxicity, corrosion of instruments, oedema and hematoma of surrounding tissues and reduction in the elastic modulus and flexural strength of dentin are some of the drawbacks that should be considered when using sodium hypochlorite [1,2]. Chlorhexidine gluconate is a broad-spectrum antimicrobial agent, and 2% chlorhexidine has been recommended for endodontic irrigation [3]. Chlorhexidine has a unique property of adsorbing to dental tissues, which enables a prolonged and extended release of the medicament. It is also found to be biocompatible with the oral tissues, by displaying low toxicity levels [3]. In vitro studies have suggested that it exhibits sustained antimicrobial activity within the root canals [3]. Chlorhexidine has thus been suggested as an endodontic irrigant owing to its unique ability to bind to dentin and its effectiveness as an antimicrobial agent due to the property of substantivity within the canal system [3]. In the recent past, soft tissue lasers like diode lasers have gained popularity for their use in dentistry. In regard to canal disinfection, the different lasers used in endodontics are CO_2_; neodymium-doped yttrium aluminium garnet (Nd:YAG); erbium-doped yttrium aluminium garnet (Er:YAG); erbium- and chromium-doped yttrium, scandium, gallium and garnet (Er:Cr:YSGG); holmium:yttrium aluminium garnet (Ho:YAG); and diode lasers [4]. Compared to the Nd:YAG laser, the diode laser enables greater water absorption within the dental tissues resulting in better penetration through the dentin, thus making it possible to act on microorganisms within the dentinal tubules [5]. Furthermore, the diode laser causes thermal photo disruptive activity in inaccessible areas of the dentin, resulting in a more substantial bactericidal impact within the root canal dentin [6]. Complete disinfection of the root canal can be challenging and unpredictable due to the complexity of the canal system and the invasion of microorganisms into the dentinal tubules, which can result in a persistent periradicular infection brought on by gram-positive, facultative anaerobes like Enterococci [7]. In failed root canal therapy, *Enterococcus faecalis* was present in 24%-77% of cases [8]. It has the proficiency to bind with dentin and persist within the niches of the torturous and inaccessible dentinal tubules [9]. The hypothesis determined that the bactericidal effect of the 810-nm diode laser along with chlorhexidine's antibacterial efficacy would be effective against the virulence factors found in *Enterococcus faecalis*. The present study aimed to compare the potency of conventionally used irrigating solutions with diode laser and diode laser along with chlorhexidine in experimentally contaminated canals with *Enterococcus faecalis.*

## Materials and methods

After obtaining approval from the Institutional Review Board at the Coorg Institute of Dental Sciences, India, the study was conducted at Azyme Biosciences Private Limited, Bangalore, India.

Preparation of specimens

Fifty mandibular first premolars extracted for orthodontic treatment were selected. The teeth selected were single rooted with intact crowns. They were decoronated at the cementoenamel junction and 3 mm from the apical portion to obtain an average middle third length of 6-7 mm. Biomechanical preparation was initiated, and the internal diameter of the canals was standardised by enlarging up to 60 size K-file. This was done to expose the dentin so that *Enterococcus faecalis* could be inoculated. After that, the specimens were autoclaved [10].

Evaluation of bactericidal activity against *Enterococcus faecalis*

*Enterococcus faecalis* was used as the study's test organism (Hi Media, Mumbai, India, ATCC 29212), which was obtained in a dried, stick form. The organism was sub-cultured, and the optical density was found to be 0.5 McFarland standard [10]. Each tooth specimen was placed in a pre-sterilised microcentrifuge tube containing 1 mL of Tryptone Soya Broth. Fifty microlitres of the sub-cultured *Enterococcus faecalis* was transferred into each microcentrifuge tube. The specimens were put into a fresh broth containing *Enterococcus faecalis* after 24 hours. All the procedures were completed in laminar flow conditions. Contamination of the specimens was carried out for a period of 27 days [10].

Disinfection method

At the end of 27 days, the content from each microcentrifuge tube was discarded. Followed by which the specimens were irrigated with 5 mL of sterile distilled water. Specimens were then randomly assigned into five groups containing 10 samples each: group I: saline, group II: 2.5% sodium hypochlorite, group III: 2% chlorhexidine gluconate solution, group IV: diode laser, and group V: diode laser + 2% chlorhexidine gluconate solution. Specimens in groups I, II and III were irrigated with the respective group irrigants with three serial rinses of 5 mL each. Specimens from group IV underwent laser disinfection. Group V was irrigated with 2% chlorhexidine gluconate solution before laser disinfection, followed by three 5 mL serial rinses. Laser disinfection was carried out using a diode laser unit emitting a wavelength of 810 nm. A 2 mm/sec pulsed mode with a 2.5 W power output was adopted. The fibre optic tip used had a diameter of 400 [4,11]. The fibre optic tip was inserted into the canal using helicoidal movements at a speed of 2 mm/sec for 5 sec, and the depth of penetration into the canal was standardised by calibrating the fibre optic tip using a William’s periodontal probe. This method was repeated four times, with a two-second gap between each one [4,11].

Evaluation of colony-forming units

Dentin shavings were taken from the coronal and apical regions of the canals using sterile Gates Glidden drills of sizes 5 and 6, respectively. Ten milligrams of dentin was harvested from each specimen which was weighed on an electronic balance [10]. These dentin shavings were then transferred to autoclaved microcentrifuge tubes containing 1 mL of Tryptone Soy Broth, after which they were incubated for 24 hours at 37°C [10]. After 24 hours, the contents of each microcentrifuge tube were serially diluted to the fifth dilution. Fifty microlitres of fractions of the fifth dilution was plated on Tryptone Agar plates, which were then incubated for 24 hours. After 24 hours, colony-forming units were counted using the classical bacterial counting technique, and the readings were tabulated [10]. The presence of *Enterococcus faecalis* in each group was determined using descriptive statistics. Inter-group analysis was done with one-way ANOVA, and pair-wise assessment was done using Scheffe’s post hoc test [10].

## Results

**Figure 1:**
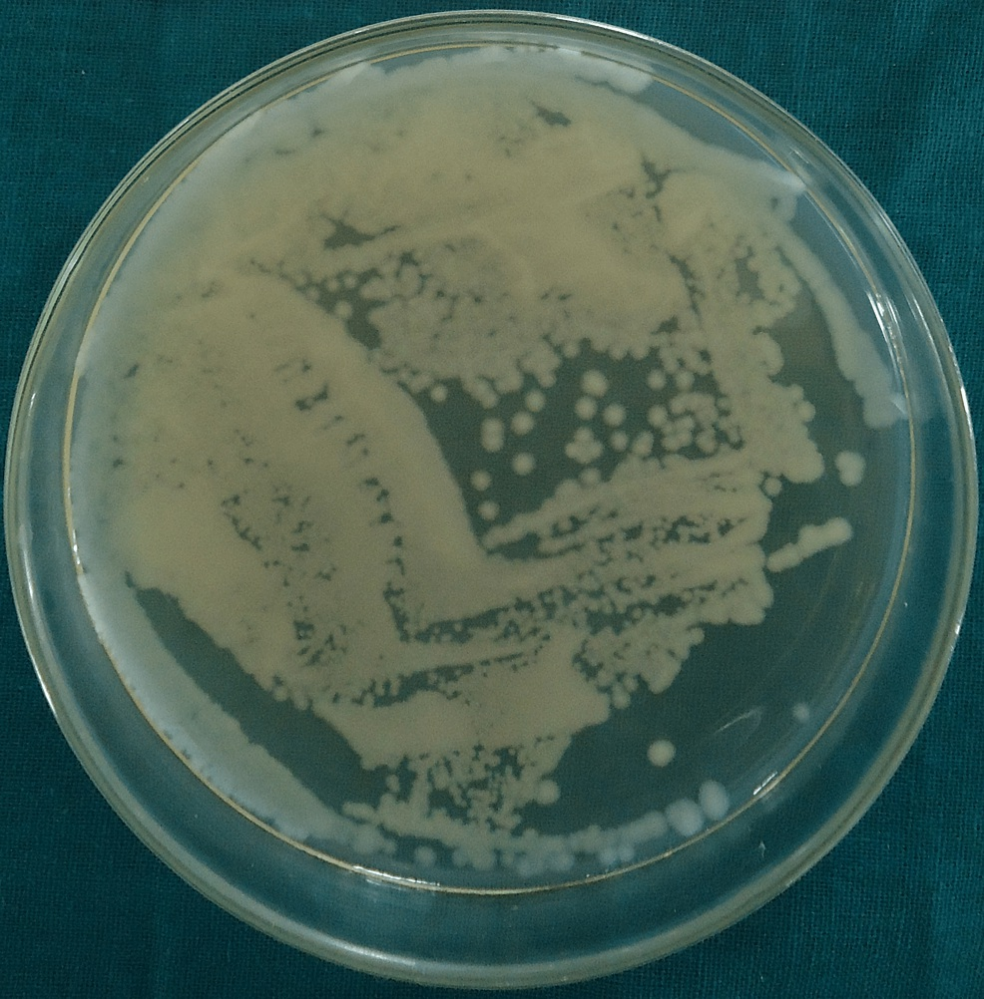
Colony-Forming Units for Group I (Saline)

**Figure 2:**
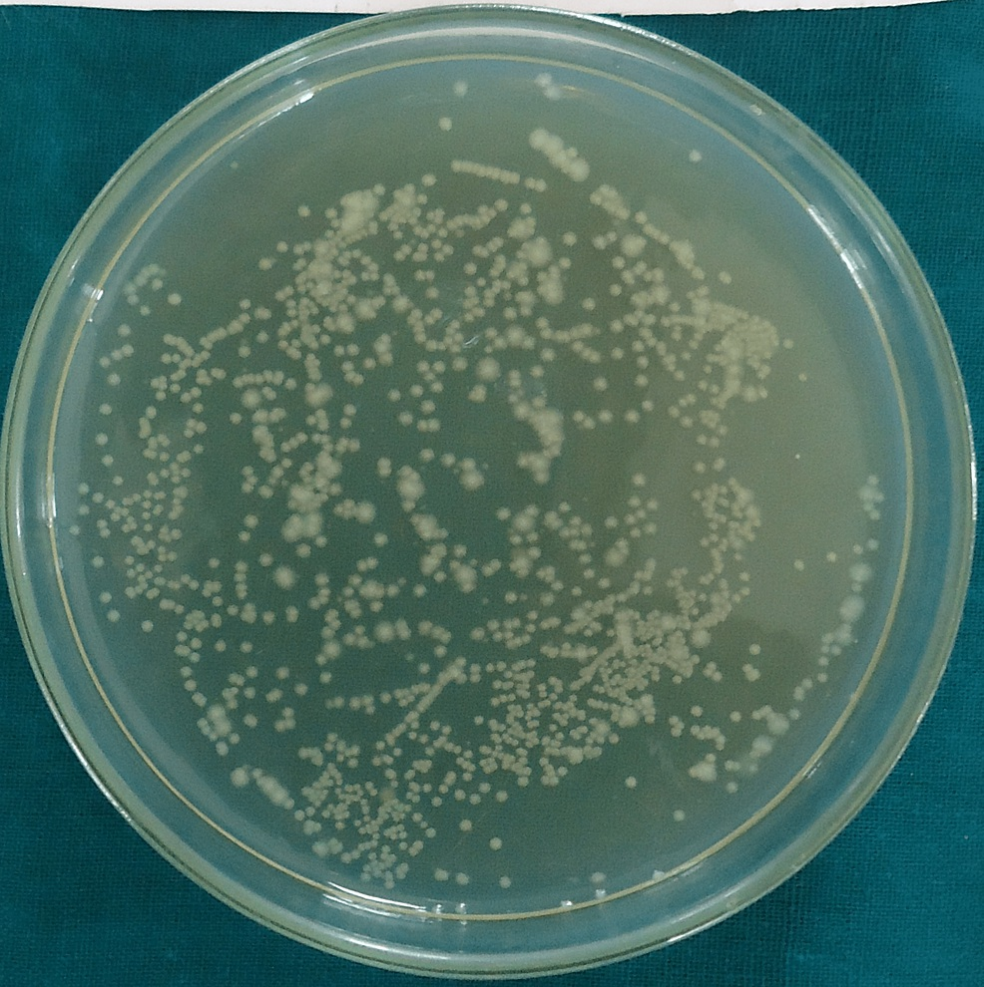
Colony-Forming Units for Group II (2.5% Sodium Hypochlorite)

**Figure 3:**
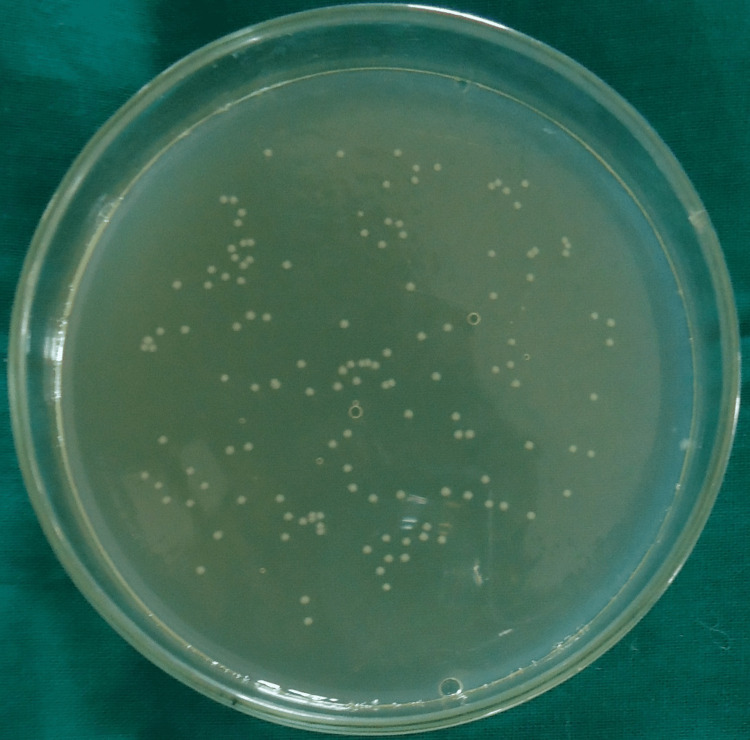
Colony-Forming Units for Group III (2% Chlorhexidine)

**Figure 4:**
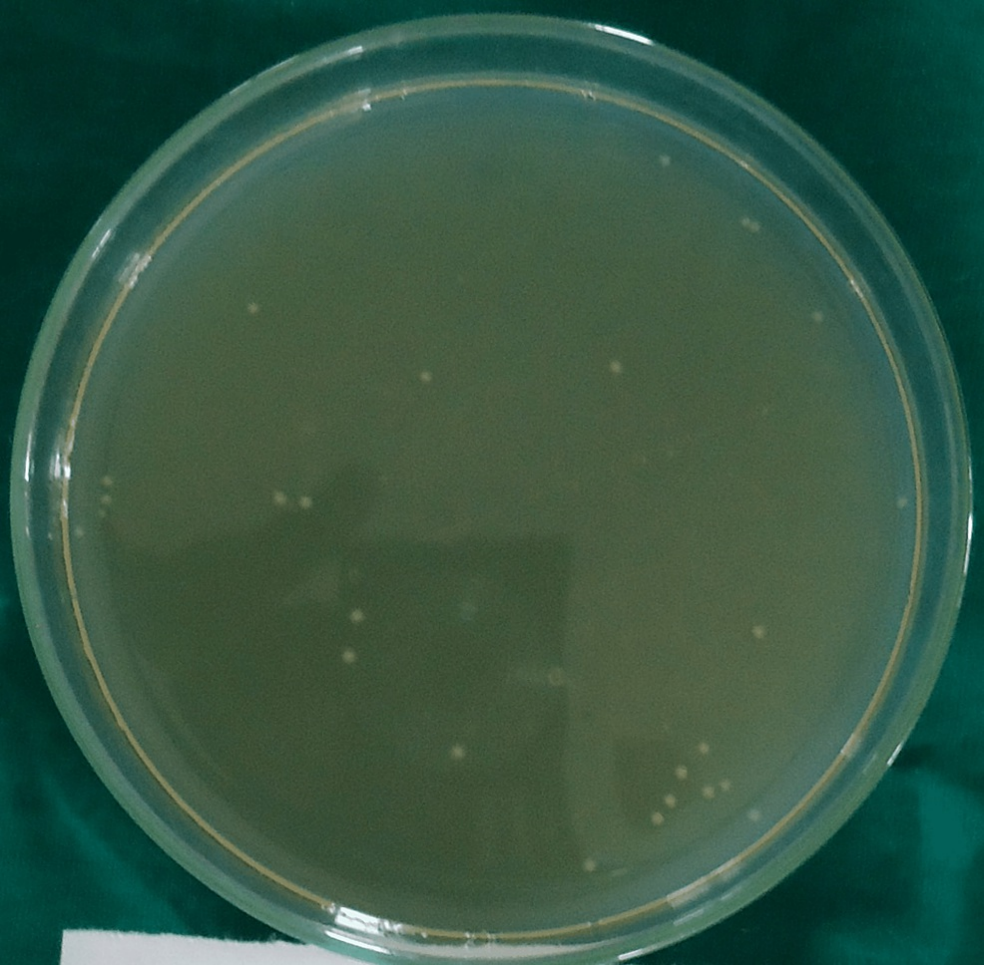
Colony-Forming Units for Group IV (Diode Laser)

**Figure 5:**
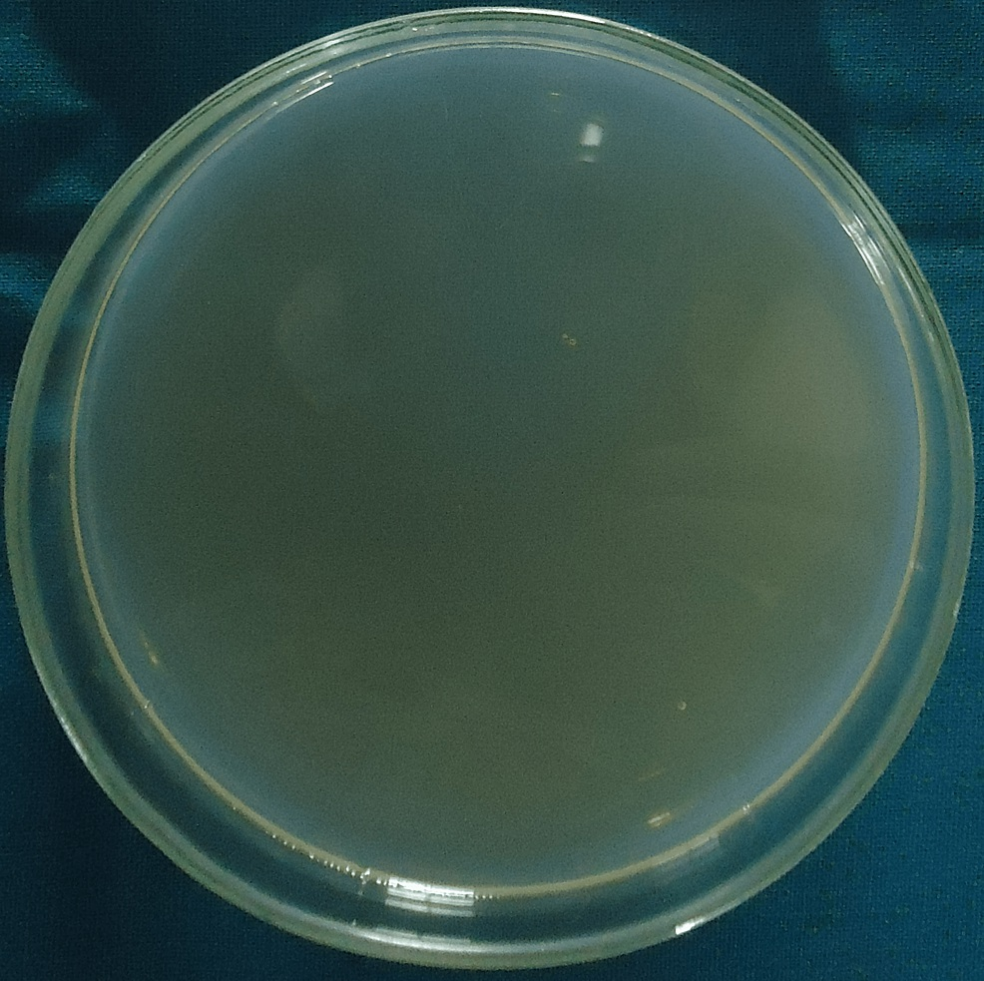
Colony-Forming Units for Group V (Diode Laser + Chlorhexidine)

Colony-forming units for group I (saline), group II (2.5% sodium hypochlorite), group III (2% chlorhexidine), group IV (diode laser) and group V (diode laser + chlorhexidine) are shown in Figures 1-5.

In all the groups, *Enterococcus faecalis* colony-forming units (CFU) were found in the collected dentin specimens that had been disinfected. The dentin shavings of the diode laser + chlorhexidine had the lowest CFU, trailed by the diode laser, 2% chlorhexidine, 2.5% sodium hypochlorite and saline (Table 1), and this variation was found to be highly statistically significant (P = 0.001) (Table 2). The post hoc test for inter-group comparison between saline, 2.5% sodium hypochlorite, 2% chlorhexidine and diode laser demonstrated a highly statistically significant difference (P = 0.001) and a non-significant difference between diode laser and diode laser with chlorhexidine (P = 0.997) (Table 3).

**Table 1:** Descriptive Statistics of the Enterococcus faecalis Count in Each Group

Group	N	Mean	Standard Deviation
I: Saline	10	1.2000×10^9^	0.00000
II: 2.5% Sodium hypochlorite	10	7.5620×10^8^	1.38232×10^8^
III: 2% Chlorhexidine gluconate solution	10	3.5880×10^8^	1.97766×10^8^
IV: 810 nm Diode laser	10	2.4000×10^7^	2.06559×10^7^
V: Diode laser with chlorhexidine	10	5.4000×10^6^	5.73876×10^6^

**Table 2:** Inter-Group Comparison Using One-Way ANOVA *Statistically significant. Df: degree of freedom.

Sum of Squares	Df	Mean Square	F	Significance
1.042×10^19^	4	2.605×10^18^	221.958	0.001*

**Table 3:** Pair-Wise Comparison Using Scheffe’s Post Hoc Test *Statistically significant. NaOCl: sodium hypochlorite, CHX: chlorhexidine.

	Saline	NaOCl	CHX	Diode Laser	Diode Laser With CHX
Saline	-	0.001	0.001	0.001	0.001
2.5% Sodium hypochlorite	0.001	-	0.001	0.001	0.001
2% Chlorhexidine gluconate	0.001	0.001	-	0.001	0.001
810 nm Diode laser	0.001	0.001	0.001	-	0.997*
Diode laser with chlorhexidine	0.001	0.001	0.001	0.997*	-

## Discussion

*Enterococcus faecalis* is an organism that, despite making up a small proportion of the flora in untreated canals, plays a major role in the aetiology of persistent periradicular lesions after root canal treatment [8]. Some of the virulence factors of *Enterococcus faecalis* include survival under prolonged periods of nutritional deprivation, altering host response and suppression of action of lymphocytes [8]. It has been documented that the biological state of the bacteria will also have an effect on the outcome of the antimicrobial treatment [12]. Starved cells survive in numbers 1,000-10,000 times higher when compared with cells in the exponential phase or stationary phase [12]. *Enterococcus faecalis* cells in this starved phase develop a biofilm on dentin, which may play a part in persistent periapical infections [12]. Siqueira et al. discovered no significant association in antibacterial properties between sodium hypochlorite concentrations ranging from 0.5% to 5.25% in root canal disinfection [13]. Although sodium hypochlorite has been found to be a potent endodontic irrigant, it has certain disadvantages, and to overcome them, alternate disinfectants are being used [13]. In this study, sodium hypochlorite had lower antibacterial potency when compared to 2% chlorhexidine which is in accordance with other studies [14-16]. Due to chlorhexidine's unique property to bind to dentin, its effectiveness as an antibacterial agent against *Enterococcus faecalis* and substantivity in the root canal system, chlorhexidine gluconate has been suggested as a root canal irrigant [17]. It has been established that the permeability of irrigants is limited as the diameter of dentinal tubules decreases [18]. Nonetheless, due to its inherent properties of light scattering and local intensity increase, laser irradiation allows light to penetrate deeper into the dentinal tubules, resulting in higher antibacterial activity [18]. In our study, we used an 810-nm diode laser, irradiated at 2.5 W, which is in accordance with several studies that have shown a reduction in bacterial load when used with similar parameters [19]. In the present study, the diode laser and diode laser + 2% chlorhexidine displayed the maximum antibacterial efficacy. The laser with chlorhexidine group had fewer colony-forming units in comparison to the laser alone group. It is plausible that irradiating the canals with a diode laser after removing debris and the smear layer using chemomechanical preparation could allow more accessibility to previously unreachable portions of the tubular network, resulting in a superior bactericidal impact on the root canal dentin [20]. The biostimulative impact of the diode laser, together with its improved bactericidal activity on the root canal dentin, suggests that it could be an ideal adjunct for root canal disinfection [21].

## Conclusions

It was determined that 2.5% sodium hypochlorite, 2% chlorhexidine, 810 nm diode laser and diode laser plus chlorhexidine exhibit antibacterial properties against *Enterococcus faecalis* under the limits of this investigation. Diode laser irradiation groups had the best antibacterial efficacy against *Enterococcus faecalis*, followed by 2% chlorhexidine, 2.5% sodium hypochlorite and finally diode laser-irradiated groups. Thus, it can be suggested that 2% chlorhexidine combined with diode laser irradiation can be used as an alternative to sodium hypochlorite in the treatment of endodontic infections. Long-term in vivo investigations, however, are required.
